# Antiviral Activity of Oroxylin A against Coxsackievirus B3 Alleviates Virus-Induced Acute Pancreatic Damage in Mice

**DOI:** 10.1371/journal.pone.0155784

**Published:** 2016-05-19

**Authors:** Bo-Eun Kwon, Jae-Hyoung Song, Hyuk-Hwan Song, Ju Won Kang, Sam Noh Hwang, Ki-Jong Rhee, Aeri Shim, Eun-Hye Hong, Yeon-Jeong Kim, Sang-Min Jeon, Sun-Young Chang, Dong-Eun Kim, Sungchan Cho, Hyun-Jeong Ko

**Affiliations:** 1 Laboratory of Microbiology and Immunology, College of Pharmacy, Kangwon National University, Chuncheon, 200–701, South Korea; 2 Agency for Korea National Food Cluster (AnFC), Iksan, Korea; 3 Department of Biomedical Laboratory Science, Yonsei University, Wonju, 220–710, Republic of Korea; 4 College of Pharmacy, Inje University, Gimhae, 621–749, South Korea; 5 College of Pharmacy, Ajou University, Suwon, 443–749, South Korea; 6 Anticancer Agent Research Center, Korea Research Institute of Bioscience & Biotechnology, Cheongju, South Korea; Hanyang University, REPUBLIC OF KOREA

## Abstract

The flavonoids mosloflavone, oroxylin A, and norwogonin, which were purified from *Scutellaria baicalensis* Georgi, significantly protected Vero cells against Coxsackievirus B3 (CVB3)-induced cell death. To investigate the in vivo antiviral activity of oroxylin A, we intraperitoneally inoculated CVB3 into 4-week-old BALB/c mice. Body weights and blood glucose levels of the mice were decreased after CVB3 infection, and these changes were attenuated by the administration of oroxylin A. Importantly, treatment of mice with oroxylin A reduced viral titers in the pancreas and decreased the serum levels of the inflammatory cytokines including interleukin-6 (IL-6) and tumor necrosis factor (TNF)-α. Additionally, the administration of oroxylin A mitigated the histological pancreatic lesions and apoptotic cell death induced by CVB3 infection and increased the levels of phospho-eIF2α in infected pancreata. The results suggest that oroxylin A may represent a potent antiviral agent against CVB3 infection.

## Introduction

Coxsackievirus B (CVB) is associated with more critical diseases, including myocarditis, pericarditis, meningitis, and pancreatitis, and can cause spastic paralysis. CVB comprises 6 serotypes denoted CVB1–CVB6. CVB3 is an important human pathogen that causes acute and chronic viral myocarditis in children and young adults and was reported to be associated with 30–50% of all myocarditis cases [1].

Oroxylin A is an *O*-methylated flavone which can be found in the medicinal plant *Scutellaria baicalensis*, which also contains baicalein, baicalin, wogonin, norwogonin, oroxylin A, and β-sitosterol [1, 2]. The pharmacological properties of oroxylin A have been widely studied. Oroxylin A has been reported to have multiple functions, including anti-inflammatory, anti-cancer [3, 4], and anti-thrombotic activities. Oroxylin A stabilizes p53 through SIRT3-mediated deacetylation via PTEN dependent manner [5], and also induces apoptosis in human cancer cell line through translocation of p53 to mitochondria [6]. Moreover, in a report associated with anti-inflammatory effect, oroxyloside, a metabolite of oroxylin A, was shown to have preventive effect against dextran sulfate sodium-induced colitis via inhibition of NF-κB pathway [7].

Recently, several studies also have shown broad-spectrum antiviral activities of extracts and compounds from *S*. *baicalensis*. Ethyl acetate and chloroform extracts of *S*. *baicalensis* were reported to show inhibitory activity against the neuraminidase enzyme of influenza virus, and this extract, as well as one of its major components, baicalein, demonstrated in vitro antiviral activity against influenza viruses including the pandemic 2009 H1N1 and seasonal 2007 H1N1 strains [8]. *S*. *baicalensis* also showed moderate or weak antiviral activity against respiratory syncytial virus (RSV), largely through wogonin and oroxylin A [9]. However, although all of these reports showed that bioactive flavones including wogonin and baicalin have antiviral activities, few studies have suggested an antiviral activity of oroxylin A [10].

In the current study, we assessed whether extracts of *S*. *baicalensis* have antiviral activity against CVB3 infection, and we found that oroxylin A and the chloroform fraction of *S*. *baicalensis*, which principally contains oroxylin A showed significant antiviral activities against CVB3 infection in vitro and in vivo. In addition, we found that the inflammatory mediators interleukin (IL)-6, chemokine (C-C motif) ligand 2 (CCL2), chemokine (C-X-C motif) ligand 1 (CXCL1), and tumor necrosis factor (TNF)-α were increased in serum after CVB3 infection in mice, but administration of oroxylin A to infected mice significantly reduced the elevation of these cytokines and chemokines. Oroxylin A showed antiviral activity associated with a reduced level of CVB3-induced cytotoxicity, which might be mediated by an increase in eIF2α phosphorylation in response to endoplasmic reticulum (ER) stress. Collectively, our results indicated that oroxylin A has the potential to be an antiviral agent against CVB3 infection.

## Materials and Methods

### Isolation of active compounds from the aerial parts of *Scutellaria baicalensis* Georgi

The dried aerial parts of *Scutellaria baicalensis* Georgi (1.2 Kg) were cut into pieces and extracted with methanol (3 × 2 L) in an ultrasonic apparatus at room temperature after evaporation of the solvent. The methanol extract was suspended in H_2_O and successively partitioned into chloroform, ethyl acetate, *n*-butanol, and water fractions after removal of the solvents under vacuum. Then, these fractions were subjected to a sulforhodamine B (SRB)-based antiviral activity assay (Table 1), and we found that the chloroform fraction had antiviral activity against CVB3. Next, the chloroform fraction was subjected to C_18_ column silica gel column adsorption chromatography (40–63 μm, 300 g) (Merck & Co., Kenilworth, NJ, USA), and eluted with a gradient consisting of methanol:water (3:7, 4:6, 6:4, 8:2, and 10:0; 2 × 500 ml). The fraction was separated on a Sephadex LH-20 column (Sigma-Aldrich, St. Louis, MO, USA) using 100% methanol, and norwogonin, oroxylin A, and mosloflavone were obtained. The structures of norwogonin, oroxylin A, and mosloflavone were identified using electrospray ionization mass spectrometry, 1H-nuclear magnetic resonance (NMR), and 13C-NMR.

**Table 1:** Antiviral activity of *S*. *baicalensis* Georgi against CVB3 in Vero cells.

	Coxsackievirus B3		
Compound	CC_50_^a^	IC_50_^b^	TI^c^
Crude methanol extract	> 50	9.53 ± 0.85	5.2
Chloroform fraction	37.5	9.76 ± 0.26	3.8
Ethyl acetate fraction	> 50	ND^d^	
Butanol fraction	> 50	ND^d^	
Water fraction	> 50	ND^d^	

Results are presented as the mean IC_50_ values ± standard deviation (SD) obtained from 3 independent experiments each carried out in triplicate.

^a^ Concentration required to reduce cell growth by 50% (μg/mL).

^b^ Concentration required to inhibit virus-induced CPE by 50% (μg/mL).

^c^ Therapeutic index = CC_50_/IC_50_

^d^ Not determined

### Cell lines and viruses

CVB3 virus (ATCC VR-30) was obtained from the Division of Vaccine Research of the Korea Centers for Disease Control and Prevention (KCDC, Cheongwon, Korea), and was propagated at 37°C in Vero cells (ATCC, Manassas, VA, USA), a kidney epithelial cell line that originated from an African green monkey. Vero cells were maintained in minimal essential medium supplemented with 10% (v/v) fetal bovine serum and 1% (v/v) antibiotic-antimycotic solution. Gibco^®^ brand antibiotic-antimycotic solution, trypsin-ethylenediaminetetraacetic acid, fetal bovine serum, and minimal essential medium were purchased from Life Technologies, Carlsbad, CA, USA, and Falcon™ tissue culture plates were purchased from BD Biosciences, San Jose, CA, USA.

### Animal model

Wild-type inbred BALB/c mice were purchased from Charles River Laboratories (via Orient Bio Inc., Sungnam, Korea). Mice were maintained under specific pathogen-free conditions in the experimental facilities at Kangwon National University. The experiments were approved by the Institutional Animal Care and Use committees of Kangwon National University (Permit Number: KW-140811-2). Female 4-week-old BALB/c mice (n = 4/group) were intraperitoneally inoculated with 1 × 10^6^ TCID_50_, the half-maximal 50% tissue culture infective dose of CVB3 in 200 μl. Groups of mice were checked for the body weight change every morning until the end of experiments. The mice were anesthesed with a mixture solution of 9:1 xelazine/ketamine and sacrificed by CO_2_ inhalation followed by cervical dislocation. BALB/C mice infected with CVB3 were administered intraperitoneally oroxylin A (10 mg/kg) for 5 days.

### SRB assay

The antiviral activities and cytotoxicities of the test extracts and compounds were evaluated by using the SRB method to measure the cytopathic effect (CPE) induced by viral infection as previously reported [11]. The Time-of-addition (TOA) assay was designed to determine the mechanism of action of antiviral compounds [12, 13]. The active compounds were then added onto the cells at 30 μg/ml either before (-1 h), during (0 h), or after (1, 2, 4, and 8 h) periods of CVB3 infection. After 48 h, SRB assay was performed.

### Replicon assay

Plasmid p53CB3-LUC [14] which contains the firefly luciferase gene in place of the P1 capsid-coding region of the CVB3 viral genome, was kindly provided by Frank J. M. van Kuppeveld (Utrecht University, Netherlands). Plasmid p53CB3-LUC was linearized by Mlu I enzyme and used for the production of CVB3 replicon RNAs by using the Ribomax large-scale RNA production system (Promega). To examine the effect of oroxylin A on the replication of CVB3 replicon, 293T cells (3.5 × 10^5^ cells/well) in a 6-well plate were transfected with 0.4 μg CVB3 replicon RNA using Lipofectamine 2000 (Invitrogen), split into 96-well plates (2 × 10^4^ cells/well), and simultaneously treated with 10 μg/ml oroxylin A or 10 μM rupintrivir (as a positive control). Eight hours after treatment, the cells were assessed for firefly luciferase activity using the One-Glo Luciferase assay kit (Promega). In the same conditions, another set of CVB3 replicon-transfected cells was assayed for cell viability using CellTiter-Glo Luminescent cell viability assay kit (Promega) [15].

### Cell viability assay

Vero cells were seeded in 96-well plates at a density of 3 x 10^4^ cells per well and incubated for 24 h. Vero cells were infected with CVB3 and treated with salubrinal at the indicated concentrations. After incubation for indicated time, cell viability was measured by SRB assay [16].

### Histology and TUNEL assay

The pancreata of mice were removed and washed with PBS before being fixed with 4% (w/v) formaldehyde for overnight. The tissues were embedded in paraffin, cut into 5 μm sections, and stained with hematoxylin and eosin (H&E). The 5 μm paraffin-embedded section were stained using an Apoptag^®^ peroxidase in situ detection kit (catalogue number S7100, Chemicon, Billerica, MA, USA) to determine the level of apoptosis by TUNEL assay according to the manufacturer’s instructions. The number of TUNEL-positive cells was counted by a pathologist using a blind test.

### Cytokine measurement (ELISA)

The levels of interleukin-6 (IL-6), tumor necrosis factor-alpha (TNF-α) and chemokine (C-C motif) ligand 2 (CCL2) were determined using a mouse ELISA Ready-SET-GO kit (ebioscience). The levels of chemokine (C-X-C motif) ligand 1 (CXCL1)/KC measured using a DuoSet^®^ mouse ELISA kit (R&D Systems, Minneapolis, MN, USA) and an ELISA MAX™ standard kit (Biolegend, Inc., San Diego, CA, USA), respectively. Experiments were performed according to each manufacturer’s instructions.

### Western blot

Rabbit anti-cytoskeletal actin (Bethyl Laboratories, Montgomery, TX, USA), Mouse anti-CVB3 VP1 (Dako, Copenhagen, Denmark), Rabbit anti-eIF2α (Cell signaling Technology, Inc.), Rabbit anti-phospho-eIF2α (Ser51) (Cell signaling Technology, Inc.) and Rabbit anti-ATF4 (CREB-2) (Santa Cruz Biotechnology, Inc.) were used. The enhanced chemi-luminescence substrate femtoLUCENT™ PLUS-HRP (G-Biosciences, St. Louis, MO, USA) was applied and images of bands were captured using an ImageQuant™ LAS 4000 Mini system (GE Healthcare Life Sciences, Little Chalfont, Buckinghamshire, UK). Quantification of band densities was performed using Image J software (NIH, Bethesda, MD, USA).

### Real-time polymerase chain reaction (PCR)

Total RNA was extracted from Vero cells and pancreata of mice using a QIAamp^®^ viral RNA mini kit (Qiagen, Limburg, Holland). Reverse transcription was performed using SuperScript™ II reverse transcriptase (Invitrogen, Grand Island, NY, USA) according to the manufacturer’s instructions. For real-time PCR analysis, the cDNA was serially diluted 10-fold and amplified using a 7500 real time PCR system (Applied Biosystems, Foster City, CA) with Power SYBR^®^ Green PCR master mix (Applied Biosystems). We used the following primers: β-actin (sense 5′-CCA TCA TGA AGT GTG ACG TGG-3′, antisense 5′-GTC CGC CTA GAA GCA TTT GCG-3′) and EV-NCR (sense 5′-CCG GCC CCT GAA TGC GG-3′, antisense 5′-ATT CTT TAA TTG TCA CCA TAA GCA GCC A-3′).

### Statistical analysis

To compare multiple groups, we carried out one-way ANOVA followed by the Tukey post hoc test using GraphPad Prism version 5 software (Graphpad, San Diego, CA, USA). Values of p < 0.05 were considered significant at a 95% confidence interval.

## Results

### *In vitro* antiviral activity of *S*. *baicalensis* against CVB3 infection

We found that the extract of *S*. *baicalensis* significantly protected against decreases in cell viability caused by CVB3 infection (Table 1). The methanol extract of *S*. *baicalensis* (IC_50_ = 9.53 μg/ml) potently inhibited CPE caused by CVB3 infection. To identify the active antiviral compounds, the methanol extract was further fractionated into chloroform, ethyl acetate, butanol, and water fractions, and we found that antiviral activity was highly retained in the chloroform fraction. The IC_50_ value of the chloroform fraction in CVB3-infected Vero cells was determined to be 9.76 μg/ml. Thus we undertook further studies to identify the antiviral compounds present in the methanol extract of *S*. *baicalensis*.

### *In vitro* antiviral activities of norwogonin, oroxylin A, and mosloflavone against CVB3 infection

**Fig 1:**
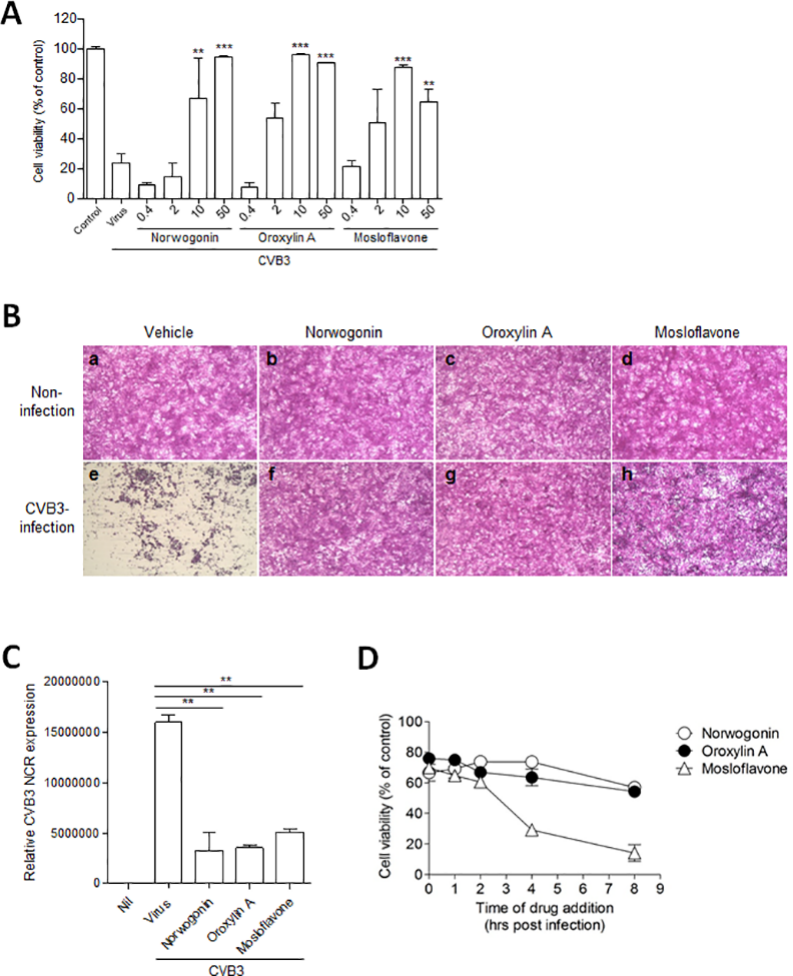
The antiviral activities of norwogonin, oroxylin A, and mosloflavone against CVB3 in vitro. (A) Antiviral activities of norwogonin, oroxylin A, and mosloflavone against CVB3 in Vero cells were measured by SRB assay. The indicated concentration of norwogonin, oroxylin A, and mosloflavone, ranging from 0.4–50 μg/ml, were added to Vero cells infected with the CCID_50_ titer of CVB3. Cells were cultured for 48 h and the antiviral activity was determined by CPE reduction assay. Absorbance values are presented as means ± SD from 3 independent experiments each carried out in triplicate. ^**^P<0.001; ^***^P<0.0001 using one-way ANOVA with Tukey’s post hoc test. (B) The effects of norwogonin, oroxylin A, and mosloflavone on CVB3–induced CPE using SRB assay. The virus-infected cells were treated with norwogonin, oroxylin A, and mosloflavone at 50 μg/mL. After incubation at 37°C in 5% CO_2_ for 48 h, the morphologies of cells were photographed under a microscope. (B-a) Non-infected cells; (B-b) non-infected cells treated with norwogonin; (B-c) non-infected cells treated with oroxylin A; (B-d) non-infected cells treated with mosloflavone; (B-e) CVB3-infected cells; (B-f) CVB3-infected cells treated with norwogonin; (B-g) CVB3-infected cells treated with oroxylin A; (B-h) CVB3-infected cells treated with mosloflavone; (C) Relative CVB3 gene expression in control, CVB3-infected, and 10 μg/ml norwogonin-, oroxylin A-, and mosloflavone-treated cells by real-time PCR. **P<0.001 using one-way ANOVA with Tukey’s post hoc test. (D) TOA effects of norwogonin, oroxylin A, and mosloflavone on CVB3 replication in Vero cells. 30 μg/mL of each compound was added either during (0 h), or after (1, 2, 4, or 8 h) virus infection. After 2 days, inhibition was evaluated by the SRB method and expressed as the inhibition rate. Percentage values represent the mean ± SD of 3 independent experiments.

After further purification, we obtained several compounds with potential antiviral activities against CVB3, including norwogonin, oroxylin A, and mosloflavone. The *in vitro* antiviral activities of these compounds were assessed (Fig 1A). All 3 compounds showed significant antiviral activity against CVB3 infection at concentrations ≥ 10 μg/ml. Although mosloflavone exhibited approximately 20% of cytotoxicity at 50 μg/ml, oroxylin A and norwogonin showed no detectable cytotoxicity against Vero cells at concentrations < 50 μg/ml, which is contradictory to several recent study showing that oroxylin A is able to induce apoptosis. However, most of them used higher concentration of oroxylin A (> 100 μg/ml) than we used, and there could be cell-type dependency [5].

The CC_50_, IC_50_, and therapeutic index (TI) values of norwogonin, oroxylin A, and mosloflavone are shown in Table 2. We analyzed the CPE inhibiting activities of norwogonin, oroxylin A, and mosloflavone on Vero cells infected with CVB3. In the absence of CVB3 infection, Vero cells treated with vehicle (Fig 1B-a) or 50 μg/ml of each compound including norwogonin (Fig 1B-b), oroxylin A (Fig 1B-c), and mosloflavone (Fig 1B-d) showed typical spread-out shapes with normal morphology. Infection with CVB3 in the absence of drug treatment resulted in a severe CPE (Fig 1B-e). Addition of norwogonin (Fig 1B-f), oroxylin A (Fig 1B-g), and mosloflavone (Fig 1B-h) to the Vero cells infected with CVB3 prevented any visible CPE from occurring. These results suggested that norwogonin, oroxylin A, and mosloflavone from *S*. *baicalensis* had significant antiviral activity against CVB3 without inducing cytotoxicity in Vero cells.

**Table 2:** Antiviral activity of Norwogonin, Oroxylin A, and Mosloflavone against CVB3 infection in Vero cells.

	Coxsackievirus B3		
Compound	CC_50_^a^	IC_50_^b^	TI^c^
Norwogonin	> 50	13.5 ± 9.83	3.7
Oroxylin A	> 50	3.17 ± 1.19	16
Mosloflavone	> 50	3.92 ± 2.15	13

Results are presented as the mean IC_50_ values ± SD obtained from 3 independent experiments each carried out in triplicate.

^a^ Concentration required to reduce cell growth by 50% (μg/mL).

^b^ Concentration required to inhibit virus-induced CPE by 50% (μg/mL).

^c^ Therapeutic index = CC_50_/IC_50_

To determine whether the increase in cell viability upon norwogonin, oroxylin A, and mosloflavone treatment in CVB3-infected cells was due to direct antiviral activity of the drug, we assessed CVB3 5' non-coding region (NCR) mRNA level 48 h after infection. The expression of 5' NCR transcript was decreased by norwogonin, oroxylin A, and mosloflavone (10 μg/ml) as compared to vehicle treatment (Fig 1C), suggesting that CVB3 replication was inhibited by norwogonin, oroxylin A, and mosloflavone.

To further characterize the antiviral activities of these compounds, we assessed their inhibitory effects on the CPE caused by CVB3 infection of Vero cells when the compounds were added at different times before, with, and after CVB3 inoculation (Fig 1D). This TOA assay is intended to determine the effects of adding the test compounds at different stages during the viral replication cycle [12, 17]. The results showed that norwogonin and oroxylin A suppressed CVB3 infection when they were added just after virus inoculation (0 h) and at the early stages after virus inoculation (1, 2, 4, and 8 h), whereas mosloflavone only showed antiviral activity when added within 2 h of CVB3 inoculation. When added at 1 h before CVB3 infection and washed out before viral infection, norwogonin, oroxylin A, and mosloflavone did not show antiviral activity. Collectively, we can presume that oroxylin A and norwogonin were effective at the early stages of viral infection and seems effective even treated 8 h after CVB3 infection, suggesting that they might be therapeutically used for CVB3 infection.

### Oroxylin A inhibits CVB3 proliferation

**Fig 2:**
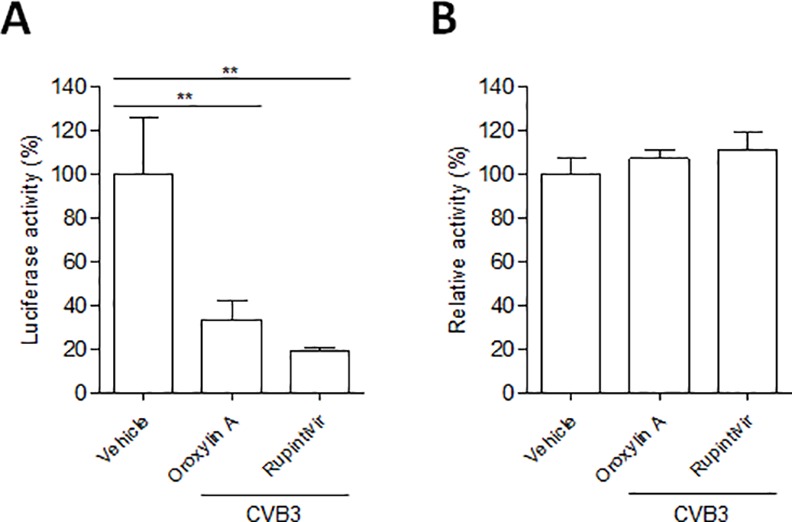
Oroxylin A inhibits the replication of the CVB3 replicon. (A) Vero cells were transfected with in vitro-transcribed CVB3-replicon RNAs, immediately treated with the indicated concentrations of oroxylin A for 8h, and then assayed for firefly luciferase activity. The luciferase activity of DMSO-treated cells was considered to be 100%. ^**^P<0.001 using one-way ANOVA with Tukey’s post hoc test. (B) In the same conditions, another set of CVB3 replicon-transfected cells was assayed for cell viability using CellTiter-Glo reagent. The activity of DMSO-treated cells was considered to be 100%.

In order to know whether oroxylin A inhibits the replication of CVB3 in infected cells, Vero cells were transfected with in vitro-transcribed CVB3-replicon RNAs, simultaneously treated with a 10 μg/ml of concentration of oroxylin A for 8 h, and then assayed for luciferase activity. Oroxylin A inhibited the replication of CVB3 replicons at a 10 μg/ml of concentration (Fig 2A). Additionally, it did not exhibited any cytotoxicity on Vero cell in 10 μg/ml concentration, which was assessed using CellTiter-Glo reagent (Fig 2B). In the same conditions, rupintrivir, a potent 3C inhibitor, which was used as positive control, showed strong antiviral activity without cytotoxic effect at 10 μM concentration.

### *In vivo* antiviral activity of oroxylin A against CVB3 infection

**Fig 3:**
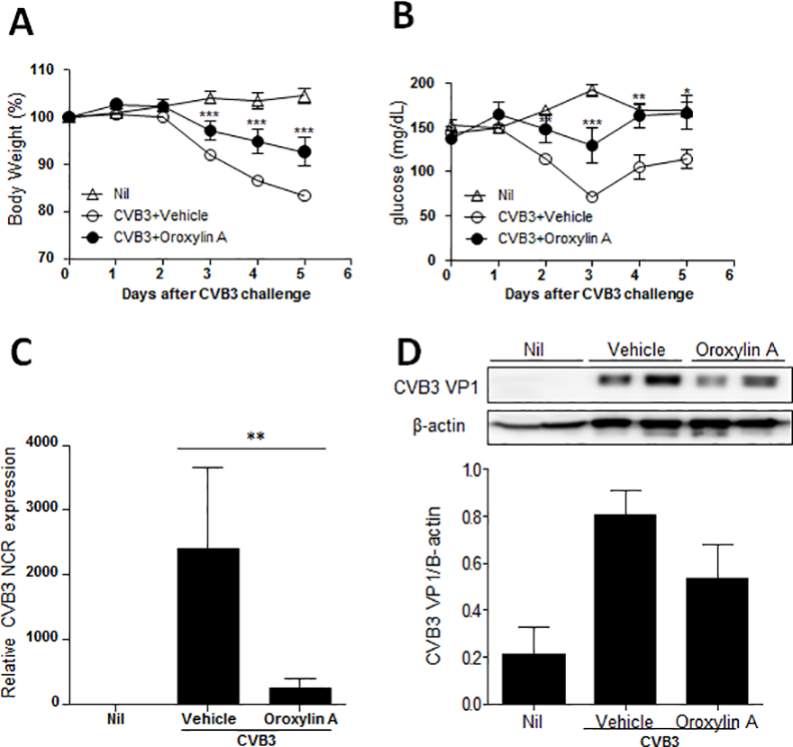
The antiviral activity of oroxylin A against CVB3 in vivo. BALB/c mice were infected with a 1 × 10^6^ TCID_50_ dose of CVB3 and given oroxylin A or vehicle. Body weights (A) and glucose levels (B) were measured for 5 days. ^*^p < 0.05, ^**^p < 0.01, ^***^p < 0.001 for CVB3 + oroxylin A versus CVB3 + vehicle, using one-way ANOVA. Virus titers of mice were determined 5 days post infection by real-time PCR (C) and western blots (D) in the pancreata. In western blot, densitometric measurement of VP1 expression is normalized to β-actin.

To ascertain the antiviral activity of oroxylin A *in vivo*, BALB/c mice were intraperitoneally injected with 1 × 10^6^ times the TCID_50_ titer of CVB3, resulting in pancreatic infection. We assessed the body weights (Fig 3A) and blood glucose levels (Fig 3B) of CVB3-infected mice. Body weights and serum glucose levels were decreased after CVB3 infection due to pancreatic damage, and these infection-induced changes were significantly attenuated by the treatment of infected mice with oroxylin A. In addition, we measured viral titers in pancreata from infected mice. Administrations of oroxylin A significantly decreased the level of CVB3 VP1 gene expression and CVB3 VP1 protein expression compared with that of infected mice administered with vehicle only (Fig 3C and 3D). These result indicated that oroxylin A exerted antiviral activity against CVB3-induced pancreatic infection *in vivo*.

### Serum cytokine and chemokine levels in mice treated with oroxylin A

**Fig 4:**
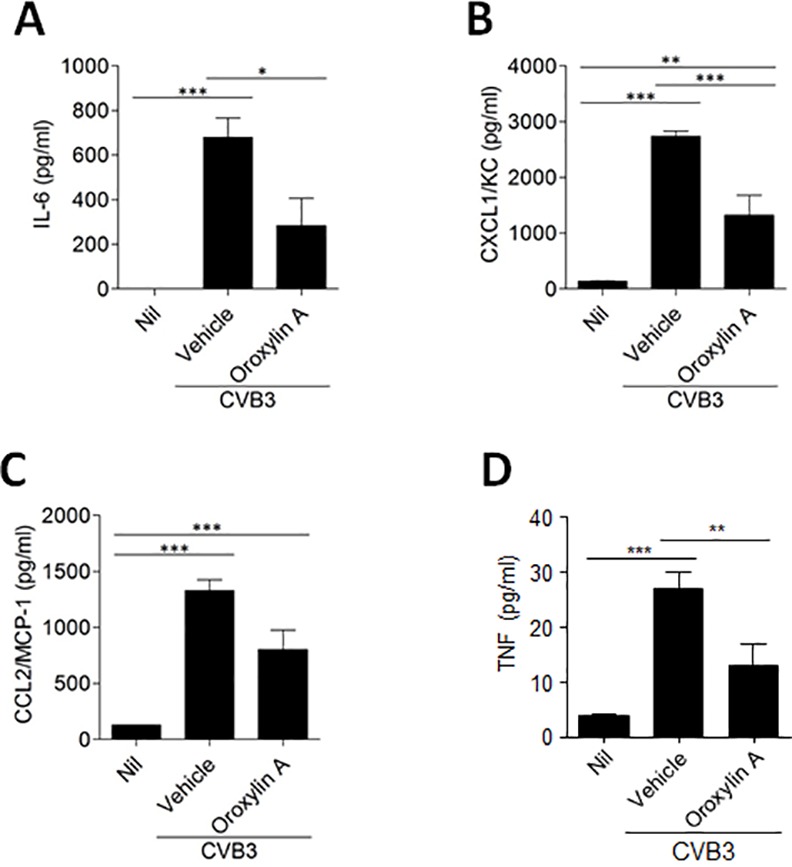
Serum cytokine and chemokine levels in mice treated with oroxylin A. Sera were collected on day 5 post infection and levels of IL-6 (A), CXCL1 (B), CCL2 (C), and TNF-α (D) were determined. ^*^P<0.01;^**^P<0.001;^***^P<0.0001 using one-way ANOVA with Tukey’s post hoc test.

Elevated levels of serum cytokines and chemokines including IL-6, CCL2, and CXCL1 have been reported to be pathological hallmarks of pancreatitis after CVB3 infection [18]. To analyze the inflammatory status of CVB3-infected mice, serum levels of IL-6, CCL2, CXCL1, and TNF-α were detected. There were minimal levels of serum IL-6, CCL2, CXCL1, and TNF-α in mice without CVB3 infection, while those infected with CVB3 had significantly increased inflammatory cytokines and chemokines on day 5 post infection (Fig 4), which might be associated with exacerbation of pancreatic inflammation. The treatment of CVB3-infected mice with oroxylin A decreased the serum levels of those cytokines and chemokines, suggesting anti-inflammatory effects of oroxylin A upon CVB3 infection.

### Oroxylin A prevents damage to the pancreas

**Fig 5:**
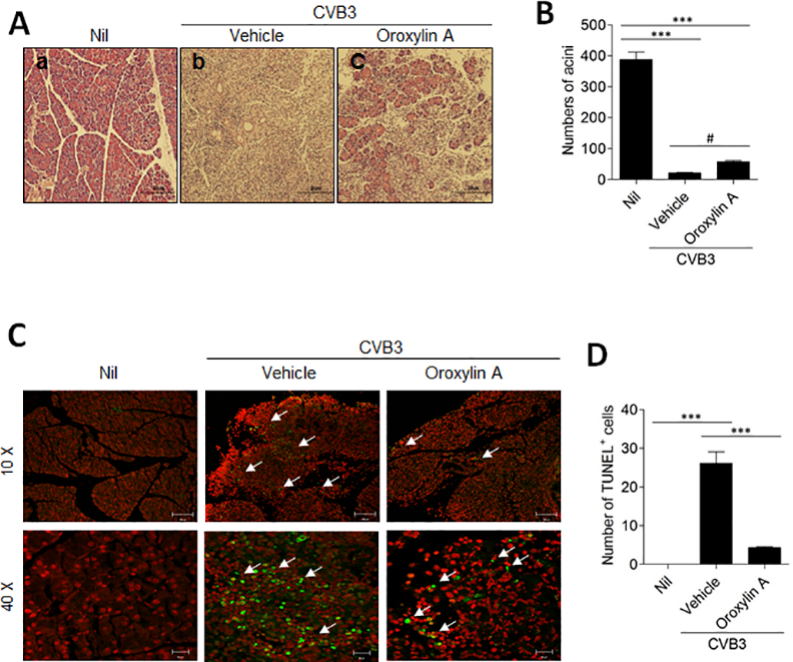
Administration of oroxylin A prevents damage to the pancreas. (A) Representative H&E staining of pancreas sections from uninfected mice (a), mice infected with CVB3 (b), and CVB3-infected mice treated with oroxylin A (c). (Scale bar = 20 μm) (B) Numbers of acini were counted from H&E images of pancreas sections. ^***^p < 0.001 as compared with the non-infected (nil) group, and ^#^p < 0.05 for CVB3 + oroxylin A versus CVB3 + vehicle (one-way ANOVA). (C) Representative image of TUNEL staining (Green fluorescence) showing the apoptotic cells in the pancreas with red fluorescence from propidium iodide-stained nuclei. Some apoptotic cells were marked with white arrow. (D) TUNEL^+^ cells were counted in pancreatic sections. ^***^p < 0.001 (one-way ANOVA).

For pathological analysis, histology sections were prepared from pancreata of mice. Uninfected pancreata were histologically normal, but those harvested 5 days after CVB3 infection showed almost complete ablation of acinar cells, as well as infiltration of inflammatory cells (Fig 5A) consistent with the decreased level of serum glucose on day 5 post infection. However, CVB3-infected mice administered with oroxylin A showed reduced histopathological abnormalities, although they still had some acinar cell hypochromicity and inflammatory cell infiltration. In addition, we confirmed that the mice administered with oroxylin A were significantly protected against the reduction in the numbers of acinar cells that occurred in the CVB3-infected, vehicle-treated mice (Fig 5B). Using TUNEL assay, we found that the pancreata of infected mice exhibited apoptotic cell death (Fig 5C and 5D). Oroxylin A treatment of CVB3-infected mice considerably decreased pancreatic apoptotic cell death and the apoptotic cells observed in those mice were found outside the pancreatic tissue rather than within it. These results supported a protective role of oroxylin A against CVB3-induced pancreatic damage.

### Oroxylin A increased phosphorylation of eIF2α

**Fig 6:**
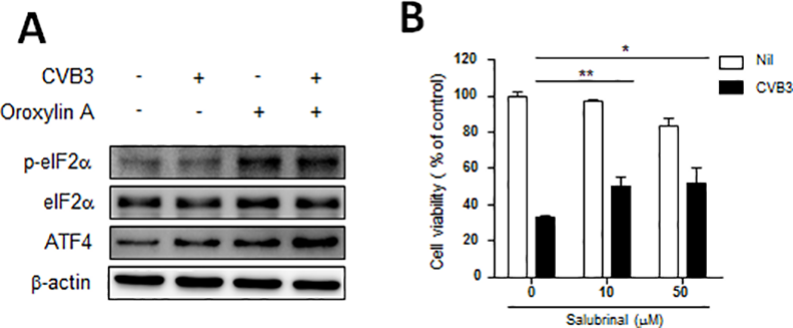
Oroxylin A enhances eIF2α phosphorylation and NF-κB signaling. Vero cells were infected with CVB3 and treated with oroxylin A (25 μg/ml) for 16 h. (A) Protein levels of phosphorylated eIF2α, unphosphorylated eIF2α, ATF4, and β-actin as measured by western blots. (B) The virus-infected cells were treated with salubrinal at the indicated concentrations. After incubation at 37°C in 5% CO_2_ for 48 h, cell viability was evaluated by SRB assay. ^*^p < 0.05, ^**^p < 0.01 (one-way ANOVA followed by the Tukey post hoc test).

Recently, it was reported that oroxylin A activated the ER stress response through the double-stranded RNA-dependent kinase (PKR)-like ER kinase (PERK)/eukaryotic translation initiation factor 2α (eIF2α)/activating transcription factor 4 (ATF4) pathway [19]. We confirmed that treatment with oroxylin A increased eIF2α phosphorylation in CVB3-infected Vero cells (Fig 6A). To ascertain the role of phosphorylated eIF2α, we assessed the effect of salubrinal, which was reported to be a blocking agent of eIF2α dephosphorylation [20], and we found that it inhibited the CVB3-induced CPE in Vero cells (Fig 6B). We confirmed that the treatment of cells with salubrinal moderately attenuated cytotoxicity induced by CVB3 infection. Collectively, these results showed that oroxylin A up-regulated eIF2α phosphorylation and attenuated CVB3-induced cell death and partially reduced acute pancreatic damage by CVB3 infection.

## Discussion

The pancreas is one of the target organs of CVB3 infection together with the liver, heart, brain, and meninges [21, 22]. Although most CVB3 infections in humans lack significant symptoms, some CVB3 infections were found to be associated with the development of type 1 diabetes with chronic inflammatory diseases of the pancreas [23], which renders CVB3 infection of the pancreas a clinically significant event [24]. Acinar and beta cells are natural primary targets of CVB3 infection [25, 26]. Infection with CVB3 induced autophagy-like vesicle formation in pancreatic acinar cells in mice [26], in which microtubule-associated protein light chain 3 conversion and large autophagic vesicles called “megaphagosomes” were observed [21]. In the current study, we confirmed that CVB3 infection in mice resulted in acute pancreatic inflammation accompanied by an increased viral load in the pancreas and increased levels of proinflammatory cytokines in serum, and these pathologic changes were significantly ameliorated by the administration of oroxylin A isolated from the medicinal plant *S*. *baicalensis*.

It was reported that some serum cytokines and chemokines were elevated during CVB3 infection [27], especially IL-6, CXCL1, and CCL2. In the current study, we measured serum cytokines and chemokines 5 days post CVB3 infection, and found significant increases in the levels of IL-6, CXCL1, CCL2, TNF-α. However, we did not detect significant changes in the levels of other cytokines including IL-4 and IL-10 (data not shown). On the basis of the elevation of inflammatory cytokines in the serum after CVB3 infection in mice, we decided to evaluate the levels of those cytokines to further assess the antiviral activity of oroxylin A against CVB3 infection *in vivo*. We hypothesized that the increased levels of CXCL1 and IL-6 could be driven by increased nuclear factor κ-light-chain-enhancer of activated B cells (NF-κB) signaling as a result of the increased ER stress caused by CVB3 infection.

Since CVB3 is a nonenveloped RNA virus that replicates in double-membrane vesicles, we can predict that CVB3 infection may influence ER-associated cellular processes. Likewise, many kinds of viruses have been shown to trigger ER stress after infection by utilizing different molecular pathways associated with the unfolded protein response (UPR) [28]. The UPR mainly involves 3 interconnected pathways centered on serine/threonine-protein kinase/endoribonuclease (IRE1)/x-box binding protein 1 (XBP1), PERK/eIF2α, and ATF6, respectively. CVB3 infection induced apoptosis in HeLa cells, and this was correlated with the induction of C/EBP homologous protein (CHOP), sterol regulatory element binding protein 1, and caspase-12. CVB3 infection activated the PERK/eIF2α and IRE1/XBP1 pathways while suppressing p58^IPK^, a negative regulator of PKR and PERK [29], in an ATF6a dependent manner [28]. Since ER stress responses were previously known to be closely linked with autophagy pathways, CVB3 might regulate the ER-autophagy pathway to prevent its autophagic degradation.

EIF2α can be phosphorylated under several circumstances including starvation, viral infection, and ER stress. Accordingly, several kinases including GCN2, PKR, and PERK could be responsible for the phosphorylation of eIF2α. On viral infection, phosphorylation of eIF2α could occur by the activation of PKR, a dsRNA-dependent protein kinase, which was initially known as an actor in antiviral response of interferons. Phosphorylation of eIF2α by PKR results in attenuation of CAP-dependent translation and induces autophagy activation, but not always ends up with cell death [30]. Interestingly, oroxylin A was reported to activate UPR in HepG2 cancer cells by the PERK/eIF2α/ATF4 pathway, but excessive activation of this pathway resulted in the activation of CHOP and induced cell death [19]. In addition, recent studies have suggested that CVB3 infection induces ER stress, which is generally caused by the accumulation of misfolded/unfolded proteins in the ER, and that CVB3 residency in ER might be closely associated with viral replication and pathogenesis. It is known that salubrinal blocks eIF2α dephosphorylation and inhibits viral replication. It was previously reported that salubrinal has antiviral activity against herpes simplex virus [31]. With a number of recent studies having reported associations between eIF2α phosphorylation and antiviral activity, it is possible that salubrinal or similar compounds may have antiviral effects via regulating the ER stress response during viral infection [20, 32].

In conclusion, the results show that administration of oroxylin A exhibited an antiviral effect against CVB3 infection and consequently decreased serum levels of inflammatory cytokines and the severity of histological lesions in mice infected with CVB3. We confirmed that oroxylin A affected the phosphorylation of eIF2α and cell death. Based on these results, we suggest that oroxylin A could be a candidate for therapeutic use as an antiviral against CVB3 infection.
